# Parents reinforce the formation of first impressions in conversation with their children

**DOI:** 10.1371/journal.pone.0256118

**Published:** 2021-08-13

**Authors:** Adam Eggleston, Cade McCall, Richard Cook, Harriet Over

**Affiliations:** 1 Department of Psychology, University of York, York, United Kingdom; 2 Department of Psychological Sciences, Birkbeck, University of London, London, United Kingdom; University of Pécs Medical School, HUNGARY

## Abstract

The tendency to form first impressions from facial appearance emerges early in development. One route through which these impressions may be learned is parent-child interaction. In Study 1, 24 parent-child dyads (children aged 5–6 years, 50% male, 83% White British) were given four computer generated faces and asked to talk about each of the characters shown. Study 2 (children aged 5–6 years, 50% male, 92% White British) followed a similar procedure using images of real faces. Across both studies, around 13% of conversation related to the perceived traits of the individuals depicted. Furthermore, parents actively reinforced their children’s face-trait mappings, agreeing with the opinions they voiced on approximately 40% of occasions across both studies. Interestingly, although parents often encouraged face-trait mappings in their children, their responses to questionnaire items suggested they typically did not approve of judging others based on their appearance.

## Introduction

Adults spontaneously attribute a wide range of traits to strangers based solely on their facial features. These first impressions include judgements about trustworthiness, honesty, competence, intelligence, aggression, and likeability [1–5]. While a wealth of spontaneous judgements have been studied, observers’ judgments appear to load on two principal dimensions often described as ‘trustworthiness’ and ‘dominance’ [1, 5]. These first impressions exert a powerful influence over behaviour. For example, individuals who appear untrustworthy are less likely to be offered jobs [6] and more likely to face harsh sentences in criminal justice situations [7, 8]. Individuals who look competent are more likely to be elected to public office [9].

Interestingly, although some first impressions may be based on ‘a kernel of truth’ [10, 11], many others appear unrelated to the true behavioural tendencies of the people being judged. For example, although observers show relatively high levels of agreement regarding which individuals appear trustworthy, these individuals are no more likely to act in prosocial ways than are members of the general population [12].

Developmental research has recently begun to investigate the origins of first impressions in young children. Cogsdill et al. [13] found that children as young as 4 were able to identify which computerised faces had been manipulated to appear ‘nice’, ‘strong’ and ‘smart’. Children’s judgments converge with those of adults and reach adult-like levels of consistency around the age of five or six in this paradigm.

Emotional expression appears to play an important role in guiding children’s reactions to others. Jessen & Grossmann [14] found that 7-month-old infants prefer to look at faces whose features seem to resemble subtle smiles rather than subtle frowns. Later in development, children use emotional expressions to guide their behaviour: five- to 12-year-old children are more likely to invest resources in an individual who is smiling than an individual who is frowning [15, 16]. The extent to which emotional expressions can be used to scaffold trait inferences continues to develop throughout childhood. Mondloch et al. [17] found that whereas adults use emotional cues to happiness and anger in order to make judgments about likely future behaviour (e.g., "would help fight dragons" vs. "would not steal your cape"), 4- to 11-year-old children do not.

Researchers agree that learning plays an important role in the acquisition of at least some first impressions [18]. Supporting this view, research has shown that there is considerable variation in first impressions across cultures [19, 20]. Further evidence comes from twin studies which demonstrate that these individual differences in trait inferences are shaped by personal experiences, rather than genes or shared environments [21]. Other work has shown that children form first impressions from cultural cues such as glasses [22]. As glasses are a relatively recent product of human history, these first impressions must be learned rather than the product of an innate mechanism.

To date, relatively little research has investigated *how* first impression are learned. Recently, however, Over & Cook [18] articulated a cultural learning perspective on the origin of first impressions. According to this view, first impressions are the result of mappings between ‘face space’ and ‘trait space’ brought about as a result of experience. Cultures consistently pair particular features of appearance with particular character traits. For example, in Western cultures villainous characters are more likely to be depicted with some kind of dermatological disorder, both in modern films [23, 24] and classic literature [25]. Likewise, depictions of princesses in Disney films consistently pair feminine features, physical beauty, and large eyes with docility and kindness [26].

According to the cultural learning model, one source of face-trait mappings is social interactions between parents and children. Parents may teach their children to make judgments about other people’s characters from their physical appearance [20]. One route by which intergenerational transmission of face-trait mappings could occur is social referencing–children may learn how to respond to strangers that vary in physical appearance by monitoring the caregivers’ non-verbal reactions to different individuals [18, 27]. Another route by which inter-generational transmission could occur is conversation. Parents may explicitly endorse or encourage particular face-trait mappings in conversation with their children [18, 20].

Here, we investigate whether parents engage in conversations with children in which they encourage their children to make inferences about other people’s characters from their physical appearance. In Study 1, we presented children with a storybook containing images of four faces–one who appeared trustworthy (high trust face), one who appeared untrustworthy (low trust face), one who appeared competent (high competence face) and one who appeared incompetent (low competence face). We gave parents the relatively open instruction ‘Talk about each of the characters shown with your child’ and recorded the conversation that resulted. Of particular interest was whether parents would ever spontaneously reference trait terms such as how kind or mean the individuals in the photograph appeared and, if so, how often. We were also interested in whether parents spontaneously made reference to subtle emotional expressions of the individuals. We also wanted to explore how discussions started and how parents responded to their child’s inferences, for example whether or not they reinforced the idea that the traits of individuals can be inferred from their appearance alone.

In addition to coding parents’ conversations with their children, we also asked parents three questions about judging people based on their appearance. These questions related to how acceptable parents found it to judge strangers based on their appearance and how confident parents were that their first impressions were accurate. Previous research found that physiognomic beliefs, the idea that psychological characteristics can be inferred from physical facial features, are relatively common and that those who more strongly endorsed physiognomic beliefs were likely to be both overconfident in their accuracy and more reliant on physical facial cues during an economic trust game [28]. We were interested in the more specific question of whether or not parents’ judgments would correlate with the extent to which they taught their children to judge individuals based on their appearance in a storybook paradigm.

We chose to investigate these questions with the parents of 5- and 6-year-old children. We chose this age group because we know that children in this age group appear to form some first impressions from appearance but their first impressions have not yet reached adult levels of consistency [13, 17]. These studies are exploratory in nature. Rather than engaging in hypothesis testing, we sought to characterise the conversations of parents and their children on these topics. The data for all studies can be found at the OSF: (https://osf.io/3d9rf/?view_only=5710f5f555ad41c094f11f930f26e091).

## Study 1

In this study, we presented parent-child dyads with a picture book containing four images. These images were of synthetic faces created using Face Gen 3.1 to appear high in trustworthiness, low in trustworthiness, high in competence and low in competence (taken from Oosterhof & Todorov [1]). We asked parents and their children to “talk about each of the characters shown”. We measured how often parents and their children referred to the apparent traits and emotions or expressions of the individuals depicted without being explicitly prompted to do so. We also coded who initiated these conversations and how often parents reinforced the face-trait mappings of their children.

## Method

### Participants

Protocols were approved by the University of York’s Psychology Ethics Committee. A total of 48 individuals participated in the form of twenty-four parent-child dyads (9 Mother-Daughter, 9 Mother-Son, 3 Father-Daughter, 3 Father-Son). Participant numbers were decided in advance based on previous research exploring parent-child interactions [29–32] Of the 24 children, 12 were 5-year-olds (12 boys, Mage: 66 months, age range = 60 to 71 months) and 12 were 6-year-olds (12 boys, Mage: 77 Months, age range = 73 to 82 months). A majority of children (20/24) were described by their parents as white British. Of the remaining 4 children, 3 were described as White/Asian and 1 was described as Indian/British. All parents (Mage = 38, SDage = 8.46) confirmed that English was both their own and their child’s primary language. Participants were recruited from a science museum in an urban centre where both oral and written consent was obtained, verbal assent was also elicited from children.

### Materials

**Fig 1:**
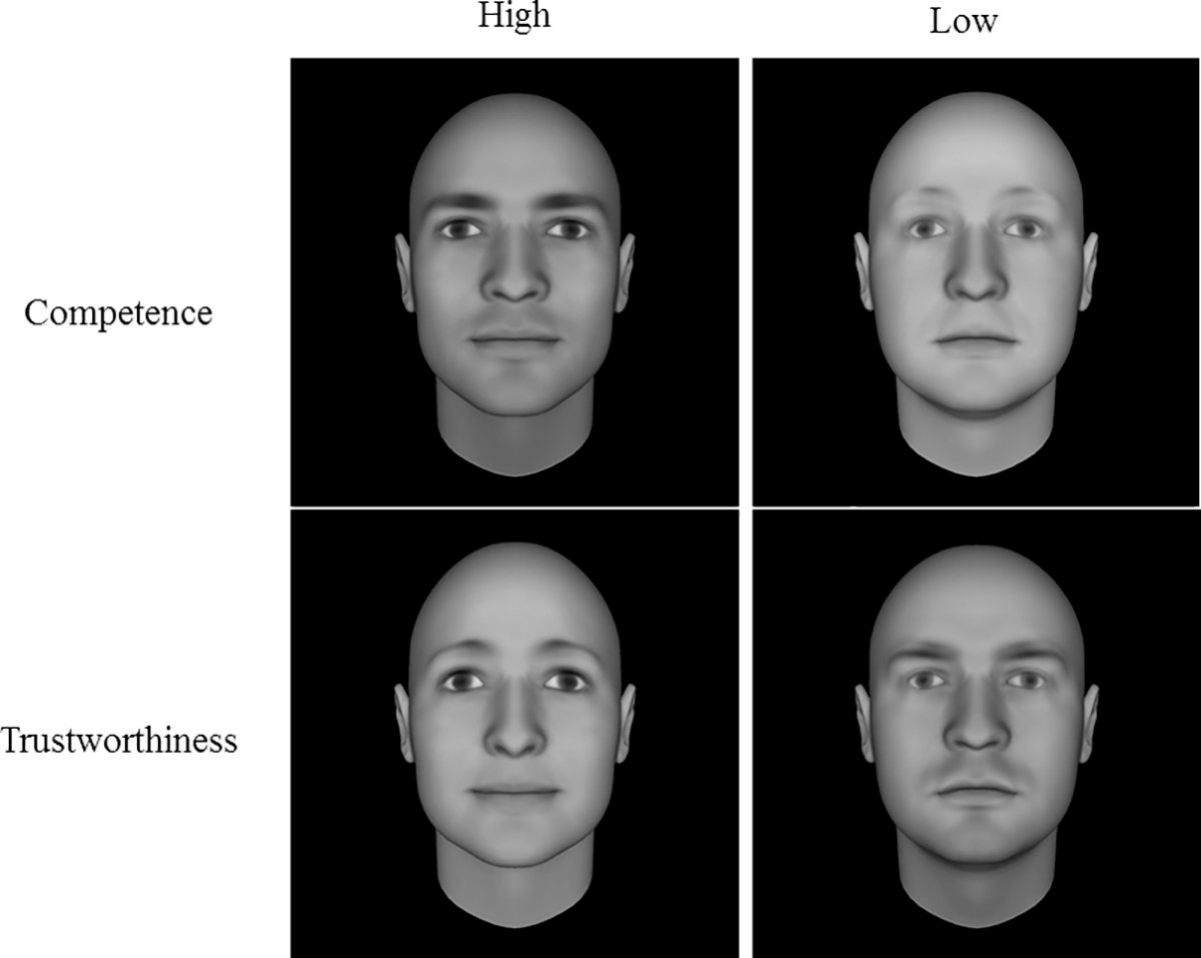
Computer generated face stimuli. All stimuli were created using Face Gen 3.1 and taken from publicly available sets of Original Computer Generated Faces, Oosterhof and Todorov [1].

The stimuli used in Experiment 1 were computer-generated face stimuli created in Face-Gen 3.1 (Oosterhof & Todorov [1])). Stimuli were chosen based on previous research suggesting that children are sensitive to apparent variations in trustworthiness and competence in these images [13]. The faces were designed to be neutral on facial expression and represent high trust, low trust, high competence and low competence. The two faces used to represent each extreme of the trait were either 3 SDs above or below the average face on the particular dimension of interest (Fig 1).

As well as the face stimuli, parents were given a three item questionnaire to complete measuring their explicit attitudes towards judging others on first impressions. Question 1: How okay or not okay do you think it is to judge someone based only their appearance?; Question 2: How okay or not okay do you think it is to teach children to judge others based only on their appearance? Both these questions were rated on a scale of 1–7 (1 = never ok, 4 = sometimes okay, 7 = always okay. Question 3: How accurate do you think you are when forming a first impression about someone else based only on their appearance? Question 3 was rated on a scale of 1–7 (1 = never accurate, 4 = sometimes accurate, 7 = always accurate). Results for the questionnaire data can be found at the end of the results section for Study 2 as data from Studies 1 and 2 were combined.

### Procedure

Participants were presented with printed versions of the stimuli formed as a book. The order of stimuli was random for each participant. The brief verbal instruction given to each parent was, “Talk about each of the characters shown with your child.” This same instruction was present on a cue card in full view throughout the experiment. Once the instruction was given the experimenter left the area to allow the participants to talk freely, these conversations were self-paced and went on for as long as the dyad desired. When finished, parents were asked to complete the 3-item questionnaire on explicit attitudes towards first impressions.

### Coding

#### Transcription

All videos were transcribed by the first author. Transcriptions started when participants engaged with the first face and ended on participants’ last reference to the picture book. Only whole words were transcribed. From these transcriptions, four aspects of the parent/child interaction were coded for; trait terms used, amount of trait discussion, emotion/expression terms used and amount of emotion/expression discussion. After identifying trait and emotion/expression discussion we then went on to code how discussion was initiated and who initiated it, as well as parents’ responses to their child’s trait talk.

#### Traits

The coding scheme used the definition of a trait supplied by Antonakis and colleagues [33] identifying traits as individual characteristics that predict attitudes, decisions, or behaviours and consequently outcomes. Every instance of a word that fit this description was coded as a trait. Examples of trait terms used were: nice, mean, trustworthy, clever, brave and adventurous.

Trait discussion was coded as the number of words used by participants in relation to a character’s traits. For instance, the below example, taken from a pair of participants, would include all words as trait discussion given that they explicitly refer to the character’s trait (kind) as well as the explanation behind the label, as in Example 1.

**Example 1**
***Parent***: So you think he might be, you think he might be a kind person?
***Child*:** Yeah.
***Parent*:** You think he might be a kind person, why do you think he might be a kind person?
***Child*:** Because he might share toys.

#### Emotions

We coded references to emotional states as well as to emotional expressions. Examples of emotion terms were happy, sad, scared, smiling, tearful and frowning.

In the same way as with trait discussion, we also coded discussion about emotions. We defined this as the number of words used by participants in relation to a character’s emotion including any further explanation, as in Example 2.

**Example 2:**
***Child*:** Is this like a, I think, I think that’s an angry face.
***Parent*:** Is it because his mouth is like that?
***Child*:** Yeah
***Paren*t:** Oh right, and what else, what else could tell you if he was angry?

#### Conversational initiation

We also sought to identify who initiated trait and emotion discussion, the parent or the child, and how these discussions were initiated. Initiations were coded in to one of three categories: questions (e.g. Do you think this person is nice), statements (e.g. This person is nice) or a combination of both (e.g. I think this person is nice, what do you think?).

#### Teaching

In order to understand whether parents teach their children face-trait mappings, we also coded whether they ever reinforced or corrected their children’s trait inferences. To achieve this, we identified each time a parent responded to their child during trait discussion and coded their response in to one of four categories: reinforcement (including agreement or repetition of the child’s response); correction (including rejection of child’s inference or an alternative suggestion); question (including where the parent questioned the child further without endorsing their response) and other responses (including changes of subject, discussion tangential to main purpose (e.g. couldn’t hear) or no follow up at all).

#### Second coding

All transcriptions were coded by the first author and second coded by a rater naïve to the rationale behind the work to assess inter-rater reliability. For the purposes of second coding, transcriptions were segmented such that each time the discussion type changed to a new topic, it was labelled as a new section in the coding sheet. These sections were then given a value of: 0 –neither trait or emotion discussion, 1 –trait discussion, 2 –emotion discussion, 3 –both trait and emotion discussion. A second coder assessed each section independently following the aforementioned coding scheme. There was near perfect agreement between the two coders’ judgements, κ = .977. The few disagreements were resolved through discussion between coders.

The number of trait and emotion terms used overall by each parent-child dyad was also assessed for inter-rater reliability. There was a strong correlation between coder’s judgements for traits (r = 1, p < .001) and emotions (r = .992, p < .001). The few disagreements between coders were resolved through discussion between coders.

For initiation of discussion, results revealed that there was near perfect agreement between the two coders’ judgements, κ = .924. Likewise, results for the inter-rater reliability analysis of parents’ trait reinforcement revealed near perfect agreement between coders, κ = .971. In all cases, the few disagreements being resolved through discussion between the coders.

## Results

To compare the number of words spoken by parents and children between conditions we used linear mixed models. These models included a fixed effect for condition (with the low trust condition set as the reference level) and a random effect for dyad to predict the number of words spoken. These models were fitted by restricted maximum-likelihood estimation in R (4.0.5) using the lme4 package (1.1.26). We also used the lmerTest package (3.1.3) to obtain anova tables for the fixed effects. The F and p-values from those tests are reported below. The estimates for the fixed and random effects for Study 1 can be found in Table A in S1 Appendix.

To test if participants were more likely to use trait words or emotion words when discussing the pictures, we used generalized linear mixed effect models to predict that binary variable (i.e., whether or not trait/emotion words were spoken at all). These models again included a fixed effect for condition and a random effect for dyad. They were fitted in R with the glmer function from the lme4 package, using a binomial (log link) as the family function. The odds ratios and random effects from these models for Study 1 are included in Tables B and C in S1 Appendix. For all models that revealed significant effects of condition, we used the emmeans package (1.5.5) for post hoc pairwise comparisons with a Bonferroni correction.

### Preliminary analyses

On average, discussions lasted for 3 minutes 15 seconds and on average parents used 319.46 words in total during the storybook task. The linear mixed model predicting the number of words spoken by parents (see Table A in S1 Appendix) did not reveal a significant effect of condition (F = 0.28, p = .843). Parents used on average 79.75 (*SD =* 52.11) words while discussing the high trust face, 78.04 (*SD =* 45.54) words while discussing the low trust face, 83.17 (*SD =* 53.7) words while discussing the high competence face and 78.5 (*SD =* 56.38) words while discussing the low competence face.

On average, children spoke 130.38 words in total during the storybook task. The linear mixed model predicting the number of words spoken by children (see Table A in S1 Appendix) did reveal a significant effect of condition (F = 2.99, p = .037). To explore the effect of condition on children’s word count, we ran post-hoc pairwise comparisons between each condition. The only contrast to emerge as significant was between the high competence and low competence conditions whereby the high competence faces elicited more words (estimate = -12.2, t(69) = -2.82, p = .037). Children spoke 33.79 (*SD =* 31.22) words while discussing the high trust face, 34.41 (*SD =* 26.7) words while discussing the low trust face, 25 (*SD =* 15.21) words while discussing the high competence face and 37.17 (SD = 31.03) words while discussing the low competence face.

### Trait terms

#### Topic of conversation

Overall, 13.3% of parent and child’s combined conversation was about the apparent character traits of the individuals depicted. Broken down individually, traits made up 14.43% of parents’ total conversation and 10.55% of children’s conversation. Illustrative examples of parents’ trait conversation are given below.

**Example 3**
***(a)***
***Parent***: Has he got a friendly face or a mean face?
***Child*:** He has, I don’t know what a cross one means.
***Parent*:** Oh, what do you think, do you think he’d be nice to you? Yeah? Okay
***(b)***
***Parent*:** Do you think they’re nice or do you think they’re grumpy?
***Child*:** Nice
***Parent*:** You think they’re nice. So do you think they’d be a helpful person if they came to talk to you?
***(c)***
***Child*:** He looks adventurous.
***Parent*:** He looks adventurous? Ah, that’s, he does, doesn’t he a bit? What else about him?
***Child*:** He looks brave.
***Parent*:** He looks brave? What makes you think he looks adventurous and brave?
***Child*:** Because, the looks of his face.
***Parent*:** The look on his face? Yeah I think I agree with you, he does look adventurous and brave doesn’t he?
***Child*:** Yeah

Other topics of conversation included references to the characters’; gender (Is this a girl or a boy do we think?), age (how old do you think he might be?), physical facial features (It’s a boy, okay, and what colour eyes are his?) and occupation (What job do you reckon this man has?).

#### Parents

The model comparing whether or not parents used trait terms in the different conditions (see Table B in S1 Appendix) did not reveal any significant effects (all p’s > .099). Discussion about traits made up 9.87% of parents’ total conversation about the high trust face, 17.67% of parents’ total conversation about the low trust face, 17.48% of parents’ total conversation about the high competence face and 12.58% of parents’ total conversation about the low competence face.

*Use of trait terms*. On average, parents used 5.71 trait terms while discussing the storybook with their children. 75% of parents used at least one trait term during the storybook task. 45.83% of parents used at least one trait term while discussing the high trust face, 58.33% of parents used at least one trait term while discussing the low trust face, 45.83% of parents used at least one trait term while discussing the high competence face and 41.67% of parents used at least one trait term while discussing the low competence face. Parents used a variety of different trait terms when describing the faces. A complete list of these trait terms, broken down by the type of face can be found in Table A in S2 Appendix.

#### Children

**Fig 2:**
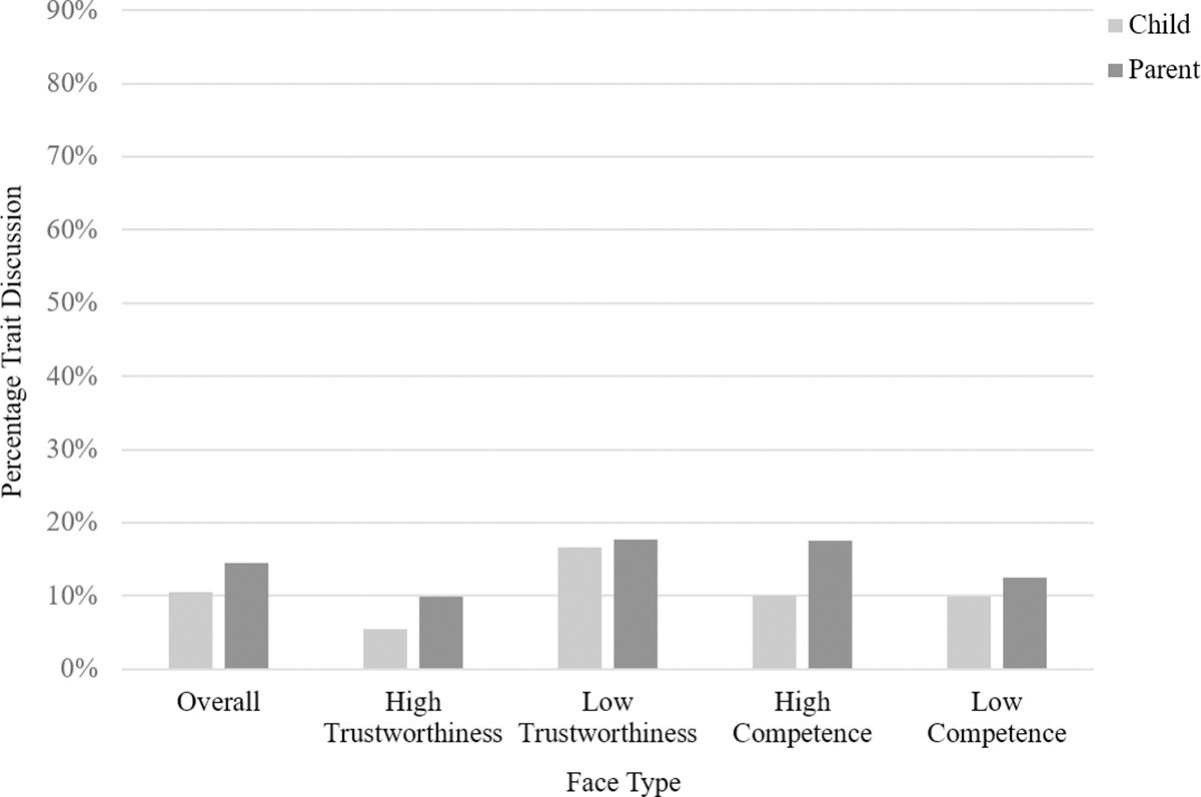
Study 1: Bar graph showing the percentage of parent and child conversation dedicated to trait talk, shown overall and separated by face type.

The model comparing whether or not children used trait terms in the different conditions (see Table B in S1 Appendix) did not reveal any significant effects (all p’s > .054). Discussion about traits made up 5.55% of children’s total conversation about the high trust face, 16.59% of children’s total conversation about the low trust face, 10% of children’s total conversation about the high competence face and 9.87% of children’s total conversation about the low competence face. A visual representation of the amount of trait discussion observed for both parents and children can be found in Fig 2.

**Fig 3:**
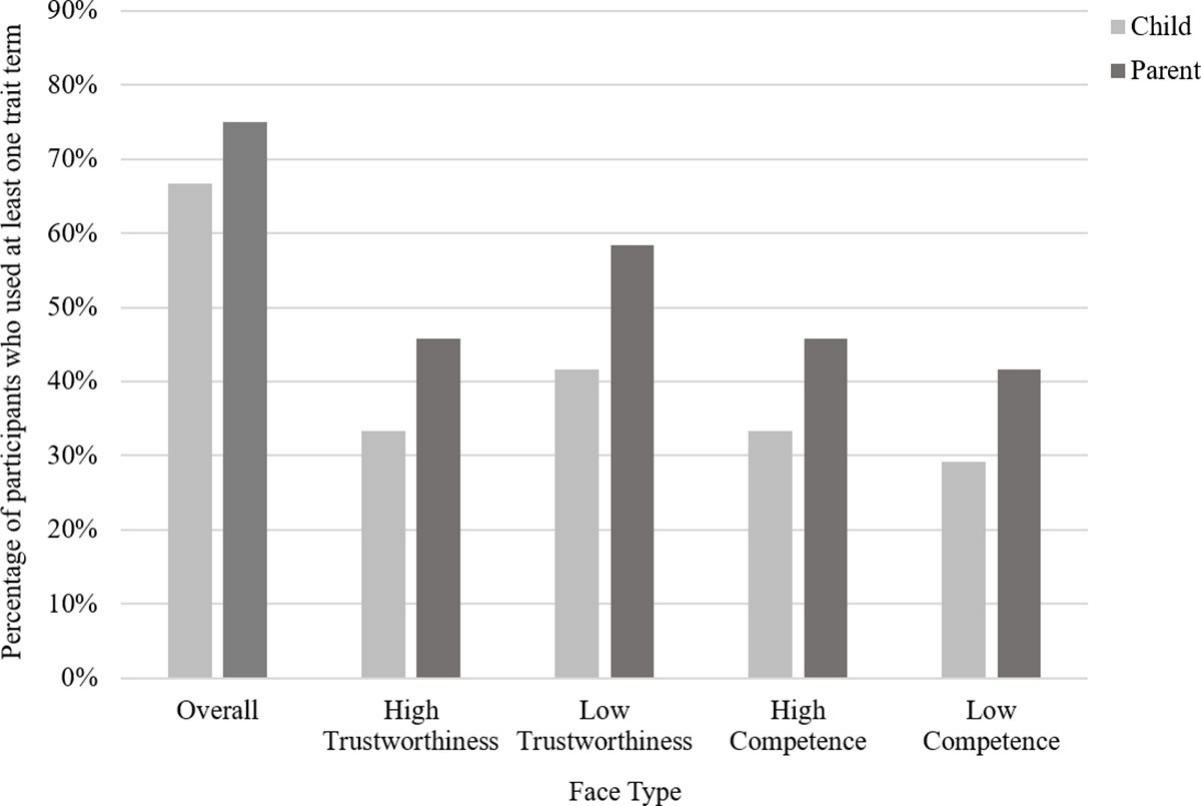
Study 1: Bar graph showing the percentage of parents (N = 24) and children (N = 24) who used at least one trait term during conversation, shown overall and separated by face type.

*Use of trait terms*. On average, children used 2.13 trait terms while discussing the storybook with their parents. A visual representation of the number of parents and children who used at least one trait term can be found in Fig 3. Overall, 66.67% of children used at least one trait term during the storybook task. 33.33% of children used at least one trait term while discussing the high trust face, 41.67% of children used at least one trait term while discussing the low trust face, 33.33% of children used at least one trait term while discussing the high competence face and 29.17% of children used at least one trait term while discussing the low competence face. Children used a variety of different trait terms when describing the faces. These terms children used broadly accord with the findings from more controlled studies (e.g. [13]). A complete list of these trait terms, broken down by the type of face can be found in Table B in S2 Appendix.

### Emotion terms

#### Topic of conversation

In addition to discussing character traits, parents and children discussed the apparent emotions of each character. Overall, combined discussion about emotions made up 9.81% of parent and child’s conversation about the faces. Broken down individually, emotion discussion made up 9.82% of parents’ total conversation and 9.78% of children’s conversation. Illustrative examples of parents’ trait conversation are given below.

**Example 4.**
***(a)***
***Child*:** Is this like a, I think, I think that’s an angry face.
***Parent*:** Is it because his mouth is like that?
***Child*:** Yeah.
***Parent*:** Oh right, and what else, what else could tell you if he was angry?
***Child*:** Red, but he isn’t red.
***Parent*:** Oh right okay.
***(b)***
***Parent*:** And are they a happy person are they a sad person?
***Child*:** Happy that guy
***Parent*:** ‘Cause they’ve got a smiling again is it?
***Child*:** Yeah
***(c)***
***Child*:** He looks a bit happier
***Parent*:** Yeah, what else makes him look happy it’s not just the smile, he can, because if I smile and go like this, what else makes him look happy then?
***Child*:** His cheeks go out wide.
***Parent*:** Yeah, anything else? He looks like he’s really happy doesn’t he? Yeah.

#### Parents

The model comparing whether or not parents used emotion terms in the different conditions (see Table C in S1 Appendix) did not reveal any significant effects (all p’s > .257). Overall, discussion about emotions made up 10.03% of parents’ total conversation about the high trust face, 13.03% of parents’ total conversation about the low trust face, 9.47% of parents’ total conversation about the high competence face and 6.79% of parents’ total conversation about the low competence face.

*Use of emotion terms*. On average, parents referred to 4.13 emotion terms while discussing the storybook with their children. 70.83% of parents used at least one emotion term during the storybook task. 41.67% of parents used at least one emotion term while discussing the high trust face, 45.83% of parents used at least one emotion term while discussing the low trust face, 41.67% of parents used at least one emotion term while discussing the high competence face and 33.33% of parents used at least one emotion term while discussing the low competence face. Parents used a variety of different emotion terms when describing the faces. A complete list of these emotion terms, broken down by the type of face, can be found in Table C in S2 Appendix.

#### Children

The model comparing whether or not children used emotion terms in the different conditions (see Table C in S1 Appendix) did not reveal any significant effects (all p’s > .138). Overall, discussion about emotions made up 11.96% of children’s total conversation about the high trust face, 11.26% of children’s total conversation about the low trust face, 6.33% of children’s total conversation about the high competence face and 8.74% of children’s total conversation about the low competence face.

*Use of emotion terms*. On average, children referred to 1.71 emotion terms while discussing the storybook with their parents. 58.33% of children used at least one emotion term during the storybook task. 41.67% of children used at least one emotion term while discussing the high trust face, 29.17% of children used at least one emotion term while discussing the low trust face, 20.83% of children used at least one emotion term while discussing the high competence face and 25% of children used at least one emotion term while discussing the low competence face. Children used a variety of different emotion terms when describing the faces. A complete list of these emotion terms, broken down by the type of face, can be found in Table D in S2 Appendix.

#### Conversational initiation

The majority of conversation about traits (73.6%) and emotions (61.4%) was initiated by parents rather than by children. Most commonly, parents introduced these topics by asking their children questions. A complete breakdown of parents’ and children’s conversational strategies can be found in Table 1.

**Table 1:** Study 1: Descriptive statistics for the number of times conversation was initiated by parent or child and the form of that initiation (question, statement or a combination) for trait and emotion discussion.

	Trait (N)		Trait (%)	Emotion (N)	Emotion (%)
Total Number of Sections	72	-		57	-
**Parent Initiated**	**53**	**73.61%**		**35**	**61.40%**
Parent Initiated via Question	45	62.50%		28	49.12%
Parent Initiated via Statement	3	4.17%		4	7.02%
Parent Initiated via Combination	5	6.94%		3	5.26%
**Child Initiated**	**19**	**26.39%**		**22**	**38.60%**
Child Initiated via Question	4	5.56%		1	1.75%
Child Initiated via Statement	15	20.83%		21	36.84%
Child Initiated via Combination	0	0%		0	0%

#### Teaching

Parents reinforced their children’s face-trait mappings, demonstrating reinforcing behaviour on 45.05% of occasions. Parents rarely directly corrected their children’s inferences (1.1% of occasions). A breakdown of parent’s teaching behaviour can be found below in Table 2.

**Table 2:** Study 1: Frequency of parents’ responses to child trait discussion by response type.

Response Type	Trait (N)	Trait (%)	Emotion (N)	Emotion (%)
Total Number of Sections	91	-	69	-
Reinforcement	41	45.05%	32	46.38%
Correction	1	1.10%	0	0%
Question	25	27.47%	22	31.88%
Change of subject	24	26.37%	15	21.74%

### Discussion

Study 1 reveals that parents engage their children in conversations about traits inferred from purely physical characteristics. Trait conversation made up just over a 10% of overall discussion about the characters in this paradigm. This provides evidence that face-trait mappings may be formed through everyday conversations between parent and child, suggesting a wealth of opportunities for these mappings to be formed and updated. Parents often led the discussion, initiating trait discussion more frequently than did their child. Interestingly, parents often initiated these conversations using information seeking questions [34]. This suggests that parents were reinforcing the view that it is possible to draw inferences about character from appearance rather than encouraging particular inferences about the specific faces depicted.

As seen in the examples provided, children were not passive learners, they initiated some trait discussion and expressed their own trait initiation. When children made trait inferences, parents expressed their agreement with them on over 40% of occasions, suggesting that parents reinforce their children’s face-trait mappings.

We also explored conversation surrounding each character’s emotional state and expression. Combined these made up over 9% of total conversation. As seen in Example 4, discussion of emotional states were often accompanied by description of the character’s expression, perhaps aiding in children’s emotion recognition ability which has been shown to increase significantly across the age range tested [35]. Related to this, other work has demonstrated that 5-year-olds ability to make trait inferences such as trustworthiness vary as a function of emotional comprehension [36] meaning that this emotion knowledge, scaffolded by parent conversation, may first be necessary before face-trait inferences can occur. Indeed, many researchers believe trait inferences to be a direct product of overgeneralisation from emotional cues [16]. Whilst the data here cannot offer causal evidence, they do point to the wealth of cultural information available to young children and one route, parent-child conversation, through which face-trait mappings could occur early in development.

## Study 2

In Study 1 we demonstrate that parents engage in conversation about traits attributed to computer generated faces. In Study 2 we are interested in the same question but seek to examine conversation about images of real faces. It is possible that parents are willing to encourage first impressions about synthetic agents who don’t really exist. When discussing real people, however, they might respond differently. By testing real-world faces we also hope to grant the task greater ecological validity, offering more of an insight into the types of conversations that could occur daily.

As in Study 1 we used faces that varied across the trustworthiness dimension. We also used faces that varied in perceived intelligence, akin to competence, perceived intelligence is interesting to explore given that inferences of intelligence may develop later that inferences of trustworthiness [22].

Again 24 parent child dyads were invited to look through a picture book containing four faces (high trust, low trust, high intelligence, low intelligence) with the instruction “talk about each of the characters shown”. Conversation was measured and is presented in the same way as Study 1.

## Method

### Participants

Protocols were approved by the University of York’s Psychology Ethics Committee. A total of Twenty-four parent/child dyads (7 Mother-Daughter, 6 Mother-Son, 5 Father-Daughter, 6 Father-Son) participated in the experiment. Of the 24 children, 12 were 5-year-olds (12 boys, Mage: 64 months, age range = 60 to 71 months) and 12 were 6-year-olds (12 boys, Mage: 77 Months, age range = 73 to 83 months). A majority of children (22/24) were described by their parents as White British. Of the remaining one was described as White and Black African and the other as Pakistani British. All parents (Mage = 37.96, SDage = 7.20) confirmed that English was both their own and their child’s primary language. Participants were recruited from a science museum in an urban centre where both oral and written consent was obtained, verbal assent was also elicited from children.

### Materials

**Fig 4:**
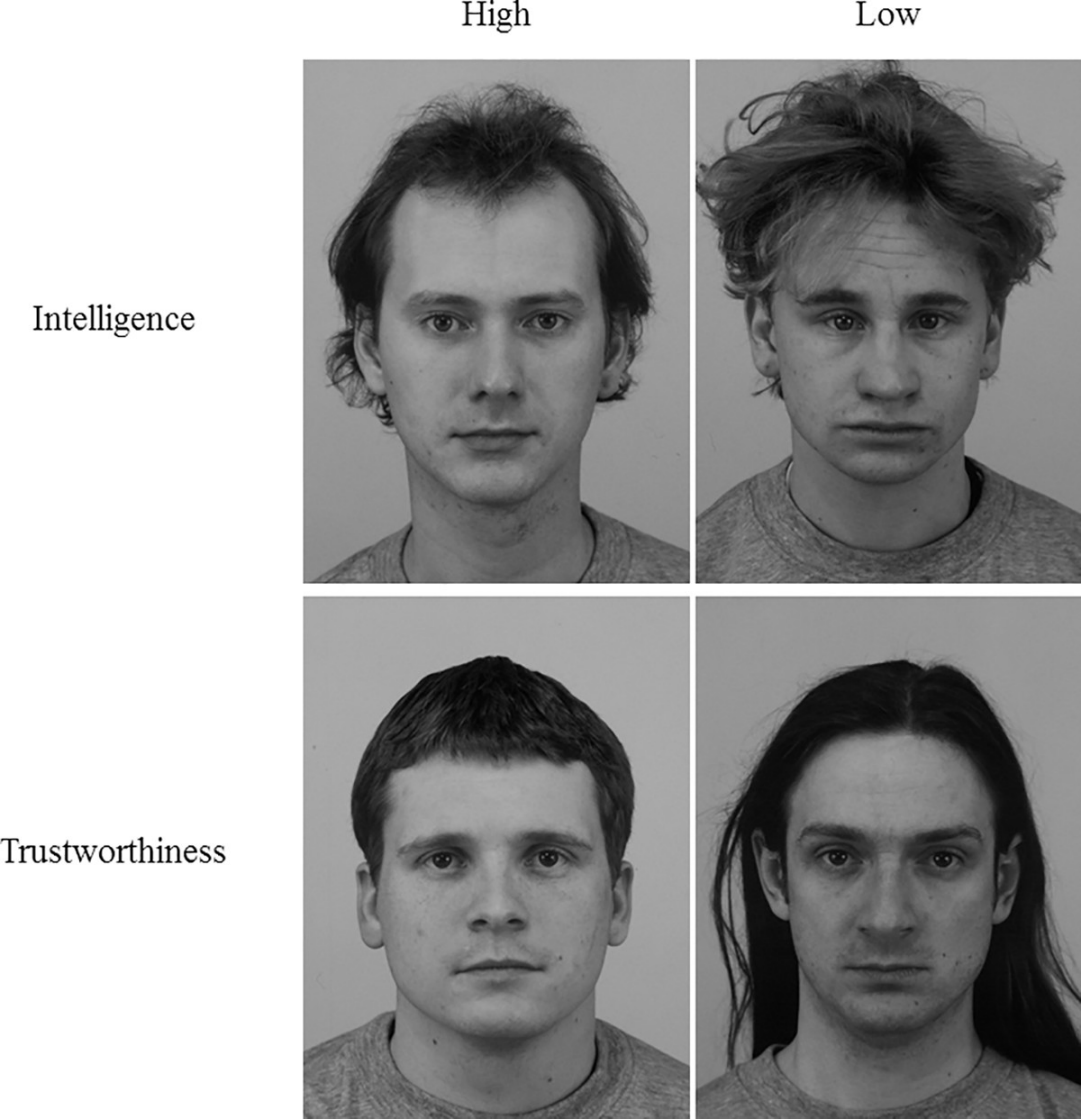
Study 2 “Real Faces”. All stimuli were taken from the Karolinska Directed Emotional Faces [37]. High Intelligence (ID: AM13), Low Intelligence (ID: AM32), High Trustworthiness (ID: AM31), Low Trustworthiness (ID: AMO3).

The stimuli used in Study 2 were taken from The Karolinska Directed Emotional Faces (KDEF) [37]. The KDEF consists of 70 faces displaying 7 different emotional expressions. For this study only expressions previously rated as emotionally neutral were included. From the original KDEF, 66 faces had been previously rated on 14 different character traits by 327 adult participants [1]. From these ratings those who ranked highest and lowest on judgements of trustworthiness and intelligence were selected to create 4 maximally dissimilar faces across the 2 dimensions, see Fig 4: High Intelligence (ID: AM13, Rating: 0.88); Low intelligence (ID: AM32, Rating -1.01); High Trustworthiness (ID: AM31, Rating: 1.04); Low Trustworthiness (ID: AM03, Rating: -1.56). All faces were presented in black and white. The same questionnaire reported in Study 1 was used in Study 2.

### Procedure

The procedure and coding were identical to that reported in Study 1.

### Second coding

All transcriptions were coded in the same way as Study 1. As in Study 1, there was near perfect agreement between the two coders’ judgements, κ = .974.There was also a strong correlation between coder’s judgements for the number of trait (r = .999, p < .001) and emotion (r = .996, p < .001) terms used by each parent-child dyad.

Agreement between coders also showed strong agreement for how parents and children initiated trait and emotion discussion, κ = .838, and parent’s trait reinforcement κ = .970. In all cases, the few disagreements between coders were resolved through discussion between coders.

## Results

The analysis plan remained identical to Study 1. To compare the number of words spoken by parents and children between conditions we used linear mixed models. The F and p-values from those tests are included in the text. The estimates for the fixed and random effects for Study 2 can be found in Table A in S3 Appendix.

To test if participants were more likely to use trait words or emotion words when discussing the pictures, we used generalized linear mixed effect models to predict that binary variable (i.e., whether or not trait/emotion words were spoken at all). The odds ratios and random effects from these models for Study 2 are included in Tables B and C in S3 Appendix.

### Preliminary analyses

On average, discussion lasted for 2 minutes 55 seconds and on average parents 300.54 words in total during the storybook task. The linear mixed model predicting the number of words spoken by parents (see Table A in S3 Appendix) did not reveal a significant effect of condition (F = 0.72, p = .545). Parents spoke 73.17 words while discussing the high trust face, 76.63 words while discussing the low trust face, 81.42 words while discussing the high intelligence face and 69.33 words while discussing the low intelligence face.

The linear mixed model predicting the number of words spoken by children (see Table A in S3 Appendix) did not reveal a significant effect of condition (F = 0.36, p = .782). Children spoke 23.04 words while discussing the high trust face, 25.63 words while discussing the low trust face, 23.46 words while discussing the high intelligence face and 23.21 words while discussing the low intelligence face.

### Trait terms

#### Topic of conversation

Overall, 14.42% of parent and child’s combined conversation was about was about the apparent character traits of the individuals depicted. Broken down individually, traits made up 14.36% of parents’ total conversation and 14.60% of children’s conversation. Illustrative examples of trait conversation are given below.

**Example 5.**
***(a)***
***Parent*:** Does he look like a nice person or a nasty person?
***Child*:** Nice person.
***Parent*:** Why does he look like a nice person? ‘Cause he looks like dad?
***Child*:** Yeah
***(b)***
***Parent*:** How do you think he looks?
***Child*:** Lazy
***Parent*:** You think he looks lazy. He looks.
***Child*:** Grumpy, grumpy, grumpy.
***Parent*:** You think he looks lazy and grumpy?
***Child*:** Yeah
***(c)***
***Parent*:** Do you think he looks like a good guy or a bad guy?
***Child*:** Bad guy.
***Parent*:** A bad guy, why do you think he looks like a bad guy?
***Child*:** Well ‘cause his face.
***Parent*:** His face, so if you saw him in a dark alleyway would you turn around and run away?
***Child*:** Yeah

#### Parents

The model comparing whether or not parents used trait terms in the different conditions (see Table B in S3 Appendix) found one significant effect. Here, the estimate of the “high intelligence” level of the condition factor was significant (Odds Ratio = .23, p = .047), suggesting a lower likelihood of using trait words in that condition. However, the post hoc comparisons between conditions did not reveal significant effects (all p’s > .284). Discussion about traits made up 11.33% of parents’ total conversation about the high trust face, 15.88% of parents’ total conversation about the low trust face, 12.38% of parents’ total conversation about the high competence face and 18.21% of parents’ total conversation about the low competence face.

*Use of trait terms*. On average, parents referred to 4.58 trait terms while discussing the storybook with their children. 75% of parents used at least one trait term during the storybook task. 41.67% of parents used at least one trait term while discussing the high trust face, 45.83% of parents used at least one trait term while discussing the low trust face, 29.17% of parents used at least one trait term while discussing the high intelligence face and 45.83% of parents used at least one trait term while discussing the low intelligence face. Parents used a variety of different trait terms when describing the faces. A complete list of these trait terms, broken down by the type of face can be found in Table A in S4 Appendix.

#### Children

**Fig 5:**
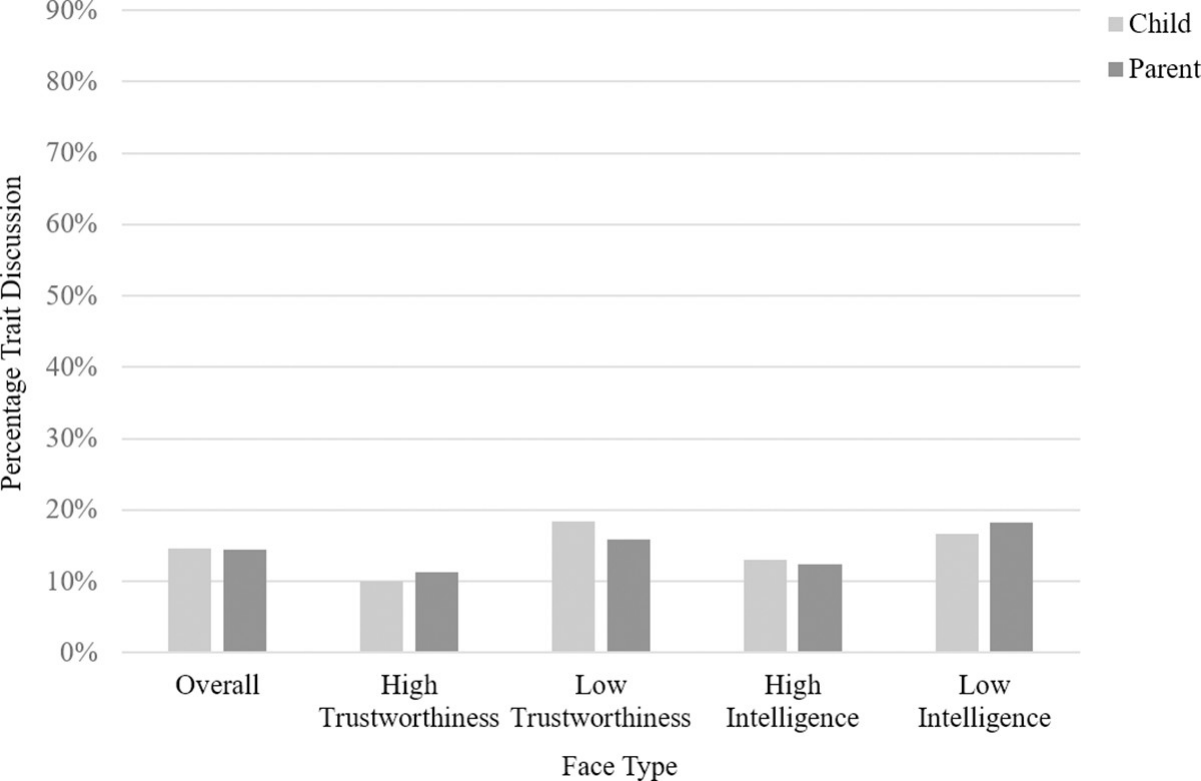
Study 2: Bar graph showing the percentage of parent and child conversation dedicated to trait talk, shown overall and separated by face type.

The model comparing whether or not children used trait terms in the different conditions (see Table B in S3 Appendix) did not reveal any significant effects (all p’s > .099). Discussion about traits made up 9.95% of children’s’ total conversation about the high trust face, 18.37% of children’s total conversation about the low trust face, 12.97% of children’s total conversation about the high competence face and 16.7% of children’s total conversation about the low competence face. A visual representation of the amount of trait discussion observed for both parents and children can be found in Fig 5.

**Fig 6:**
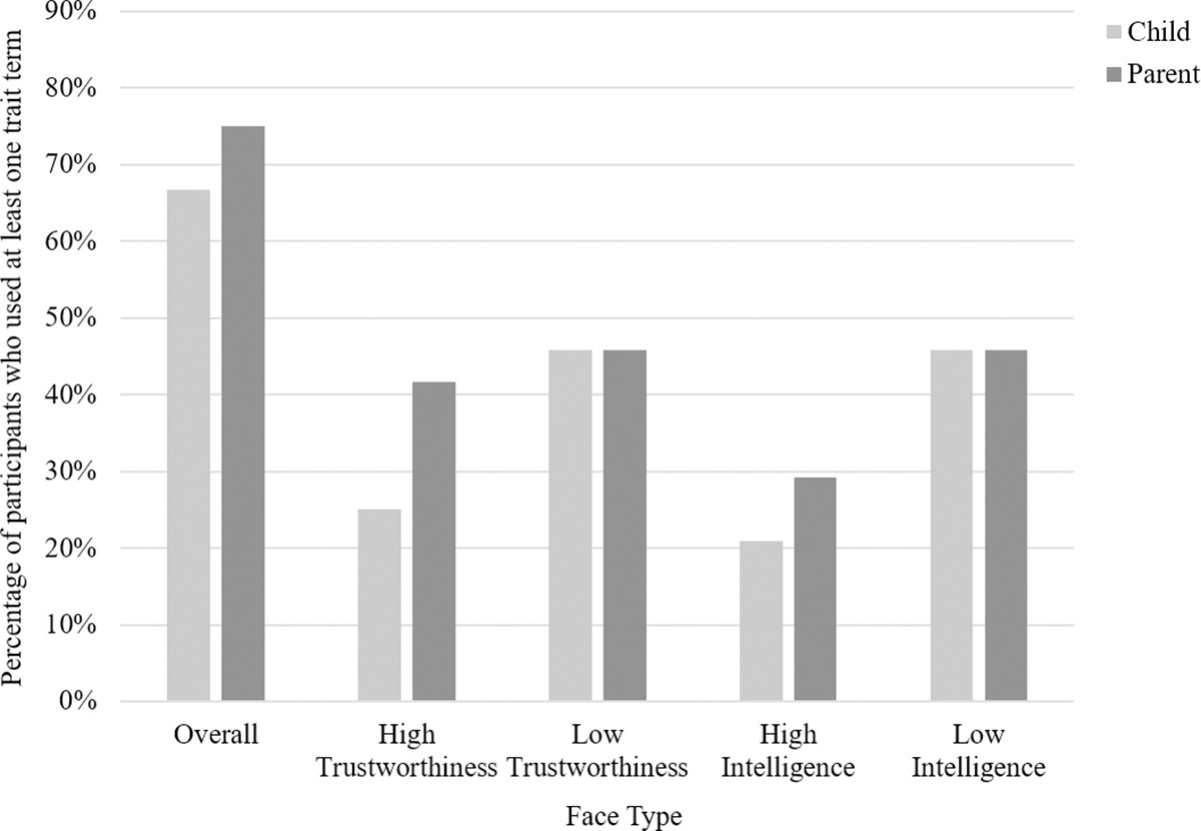
Study 2: Bar graph showing the percentage of parents (N = 24) and children (N = 24) who used at least one trait term during conversation, shown overall and separated by face type.

*Use of trait terms*. On average, children referred to 2.04 trait terms while discussing the storybook with their parents. A visual representation of the number of parents and children who used at least one trait term can be found in Fig 6. Overall 66.67% of children used at least one trait term during the storybook task. 25% of children used at least one trait term while discussing the high trust face, 45.83% of children used at least one trait term while discussing the low trust face, 20.83% of children used at least one trait term while discussing the high intelligence face and 45.83% of children used at least one trait term while discussing the low intelligence face.

Children used a variety of different trait terms when describing the faces. The terms children used broadly accord with the findings from more controlled studies (e.g., [13]). A complete list of these trait terms, broken down by the type of face can be found in Table B in S4 Appendix.

### Emotion terms

#### Topic of conversation

In addition to discussing character traits, parents frequently discussed emotions with their children. This fits with previous research suggesting that first impressions are strongly influenced by emotional cues [16, 38, 39].

Overall, combined discussion about emotions made up 16.42% of parent and child’s conversation about the faces. Broken down individually, emotion discussion made up 16.57% of parents’ total conversation and 16% of children’s conversation. Illustrative examples of parents’ trait conversation are given below.

**Example 6**
***(a)***
***Child*:** He’s sad
***Parent*:** He’s sad, why do you think he’s sad?
***Child*:** Because his mouths going down.
***Parent*:** His mouths going down, does anything else make him look sad or is it just his mouth?
***Child*:** The mouth.
***Parent*:** Just his mouth, okay
***(b)***
***Child*:** He looks a bit sad.
***Parent*:** He looks sad? Aww, why do you think he might be sad?
***Child*:** Because nobody’s playing with him.
***Parent*:** Nobody’s playing with him?
***(c)***
***Parent*:** And do you think he’s happy, sad or angry or?
***Child*:** He looks a bit sad and angry.
***Parent*:** Sad and angry, I think so too. Because he’s not smiling is he?

#### Parents

The model comparing whether or not parents used emotion terms in the different conditions (see Table C in S3 Appendix) revealed significant effects. Here, the estimates of the “high intelligence” and “high trustworthiness” levels of the condition factor were significant (Odds Ratio = 7.62, p = .045 and Odds Ratio = 19.11, p = .010), suggesting a greater likelihood of using trait words in those condition. However, the post hoc comparisons between conditions did not reveal significant effects (all p’s > .057). Discussion about emotions made up 19.93% of parents’ total conversation about the high trust face, 11.8% of parents’ total conversation about the low trust face, 17.5% of parents’ total conversation about the high intelligence face and 17.19% of parents’ total conversation about the low intelligence face.

*Use of emotion terms*. On average, parents referred to 7.46 emotion terms while discussing the storybook with their children. 83.33% of parents used at least one emotion term during the storybook task. 70.83% of parents used at least one emotion term while discussing the high trust face, 45.83% of parents used at least one emotion term while discussing the low trust face, 66.67% of parents used at least one emotion term while discussing the high intelligence face and 58.33% of parents used at least one emotion term while discussing the low intelligence face.

Parents used a variety of different emotion terms when describing the faces. A complete list of these emotion terms, broken down by the type of face, can be found in Table C in S4 Appendix.

#### Children

The model comparing whether or not children used emotion terms in the different conditions (see Table C in S3 Appendix) revealed a significant effect. Here, the estimate of the “high trustworthiness” level of the condition factor was significant (Odds Ratio = 23.51, p = .009), suggesting a greater likelihood of using trait words in that condition. However, again, the post hoc comparisons between conditions did not reveal significant effects (all p’s > .056). Discussion about emotions made up 19.17% of children’s’ total conversation about the high trust face, 13.5% of children’s’ total conversation about the low trust face, 17.23% of children’s’ total conversation about the high intelligence face and 14.36% of children’s’ total conversation about the low intelligence face.

*Use of emotion terms*. On average, children referred to 2.75 emotion terms while discussing the storybook with their parents. 58.33% of children used at least one emotion term during the storybook task. 50% of children used at least one emotion term while discussing the high trust face, 37.5% of children used at least one emotion term while discussing the low trust face, 41.67% of children used at least one emotion term while discussing the high intelligence face and 37.5% of children used at least one emotion term while discussing the low intelligence face.

Children used a variety of different emotion terms when describing the faces. A complete list of these emotion terms, broken down by the type of face can be found in Table D in S4 Appendix.

#### Conversational initiation

As in Study 1, the majority of conversation about traits (56.92%) and emotions (64.94%) was initiated by parents rather than by children. Most commonly, parents introduced these topics with questions. A breakdown of how trait and emotion discussion was initiated by participants can be found below in Table 3.

**Table 3:** Study 2: Descriptive statistics for the number of times conversation was initiated by the parent or child and the form of the initiation (question, statement or a combination of both) for trait and emotion discussion.

	Trait (N)	Trait (%)	Emotion (N)	Emotion (%)
Total Number of Sections	65	-	77	-
**Parent Initiated**	**37**	**56.92%**	**50**	**64.94%**
Parent Initiated via Question	29	44.62%	36	46.75%
Parent Initiated via Statement	3	4.62%	8	10.39%
Parent Initiated via Combination	5	7.69%	6	7.79%
**Child Initiated**	**28**	**43.08%**	**27**	**35.06%**
Child Initiated via Question	1	1.54%	0	0.00%
Child Initiated via Statement	26	40%	27	35.06%
Child Initiated via Combination	1	1.54%	0	0%

#### Teaching

Parents reinforced their child’s face-trait mappings, demonstrating reinforcing behaviour on 44% of occasions. Parents rarely directly corrected their child’s inferences (3% of occasions). Descriptive statistics characterising parents’ responses to children’s trait discussion can be found below in Table 4.

**Table 4:** Study 2: Frequency of parents’ responses to child trait discussion by response type.

Response Type	Trait (N)	Trait (%)	Emotion (N)	Emotion (%)
Total Number of Sections	100	-	103	-
Reinforcement	44	44%	50	48.54%
Correction	3	3%	4	3.88%
Question	24	24%	34	33.01%
Change of subject	29	29%	15	14.56%

### Discussion

As in Study 1 we find that over 10% of parent-child conversation centred around each character’s perceived traits. Given the lack of contextual or behavioural information regarding each character, we can assume that these trait inferences are derived from each character’s physical appearance. Providing at least some evidence that parents encourage face-trait mappings to be formed through everyday conversation. This extends upon the findings from Study 1 as we presented participants with real faces, a situation more likely to reflect day-to-day reality for the parent and child. As in Study 1, when their children voiced trait inferences from appearance, their parents often reinforced them. Together these behaviours demonstrate a plausible route through which face-trait mappings may be formed and reinforced through everyday conversation. This extends upon the findings from Study 1 as we presented participants with real faces, a situation more likely to reflect day-to-day reality for the parent and child.

The pattern in responses we saw in Study 1 for emotion discussion seem to be reflected in Study 2 with both parents and children describing emotional states and expressions in relation to each other. This corresponds with previous research suggesting that first impressions from appearance is closely tied to emotion understanding [40].

## Combined questionnaire data

### Results

#### Parents’ judgments about the acceptability of forming first impressions from appearance cues

In both studies we asked parents how acceptable they found it to form first impressions of other people’s characters from their appearance and how acceptable they found it to teach their children to form first impressions of other people’s characters from their appearance. We combined the data from Studies 1 and 2 in order to better understand parents’ answers to these questions. In general parents, judged it to be unacceptable to judge individuals based solely on their appearance. On average participants responded to the question, ‘How okay or not okay do you think it is to judge someone based only their appearance?’ with a mean score of 2.58 (Mode = 1, SD = 1.49). A one-sample t-test confirmed that this score was significantly lower than the possible middle score (4), *t*(47) = -6.61, *p* = < .001, *d* = -0.95. However, scores given ranged from 1 to 7, indicating that parents varied considerably in how acceptable they found judging other people on the basis of their appearance.

Parents also found it unacceptable to teach their children to judge the character of other people based on their appearance. On average participants responded to the question, ‘How okay or not okay do you think it is to teach children to judge others based only on their appearance?’ with a mean score of 2.56 (Mode = 1, SD = 1.61). A second one-sample t-test again confirmed that this score was significantly lower than the possible middle score (4), *t*(47) = -6.19, *p* = < .001, *d* = -0.89. Again there was a wide variability in parents’ responses with scores ranging from 1 to 7, indicating that parents varied considerably in how acceptable they found it to teach their children to treat others on the basis of their appearance. Not surprisingly, parents answers to questions 1 and 2 were highly correlated with each other–parents who thought it acceptable to judge strangers based on appearance also thought it was okay to teach their children to do so, *r* = .73, *p* < .001.

#### Parents’ impressions of their own accuracy in forming first impressions

We also asked parents how accurate they felt their own first impressions were. In general, parents were not highly confident in their ability to form accurate first impressions of others’ characters from their appearance. On average participants responded to the question, ‘How accurate do you think you are when forming a first impression about someone else based only on their appearance?’ with a mean score of 3.56 (Mode = 4, SD = 1.46). A final one-sample t-test confirmed that this score was significantly lower than the possible middle score (4), *t*(47) = -2.08, *p* = < .043, *d* = -0.30. Scores given ranged from 1 to 7, indicating that parents varied considerably in how confident they were that their judgments are accurate.

Scores from questions one and two were combined to create an overall score assessing parents’ belief in the acceptability of forming first impressions from appearance cues. We found a significant relationship and moderate correlation between parent’s belief in the acceptability of first impressions and their confidence that their first impressions were accurate, *r* = .36, *p* = .013.

#### Associations between parental attitudes and behaviour

Interestingly, parental attitudes did not correlate with the actual extent of parental teaching about first impressions in conversation with their children. Parents overall belief in the acceptability of forming first impressions from appearance cues did not correlate with either the number of trait terms parents used in conversation with their children nor the percentage of words used to discuss a character’s traits, (all *p*s>.773). Likewise, parents’ confidence in their own first impressions did not correlate with their use of trait terms nor the percentage of words used to discuss a character’s traits (*p*s>.505). Although these results must be interpreted with considerable caution due to the modest sample size, they suggest that there is not a strong relationship between parents’ explicit attitudes about the acceptability of judging people on appearance and their actual tendency to teach associations between appearance and character to their children.

### Discussion

Questionnaire data revealed that parents generally think it is unacceptable to judge others based off their physical appearance. However, responses revealed that opinion varied widely when considering whether forming impressions from appearance is an acceptable and worthy pedagogical goal. In line with previous research, those who did endorse judging others on their appearance were also more confident that their first impressions were accurate [28].

Comparing parents’ questionnaire responses to their task performance revealed that these explicit opinions did not influence their actual interactions, at least in this paradigm. Interestingly, parents who refused to endorse judgments based on first impressions were just as likely to engage in conversation about traits based purely on physical features.

## General discussion

Across two studies we aimed to investigate the important question of how first impressions may be learned. Previous research adopting a cultural learning perspective has suggested one possible way through which the inter-generational transmission of face-trait mappings could occur is through parent led conversation. In support of this, these data seem to show that parents do sometimes engage their children in conversation about the character traits attributed to unfamiliar individuals on the basis of their physical appearance. Parents engaged in this type of conversation both when discussing computer generated faces (Study 1) and real world (Study 2) faces. In line with our assumption that face-trait mappings are facilitated through parent-led conversation we found that, across both studies, parents tended to initiate these conversations, often encouraging their child to make trait inferences through the use of questions. Interestingly for our purposes, parents did this even though no explicit instruction was given to talk about the personalities of the individuals depicted. Taken together, these studies suggest that children are regularly exposed to social situations that could plausibly play a role in teaching them that it is possible to judge others’ character from their appearance [18, 41].

Further analysis of our data suggest that parents explicitly teach their children face-trait mappings, reinforcing the inferences children make approximately 40% of the time across both studies. These data suggest that, at least by the age of five, children have substantial opportunities to socially learn the face-trait mappings common within their culture. It is plausible that parental teaching is one mechanism through which children learn first impressions that are common within their culture even when they lack validity–i.e., they do not reflect the actual character traits of the individuals being judged [18].

It is interesting to note that children were active participants in the conversations we recorded, commenting on the apparent character traits of the individuals depicted themselves. This accords with previous research suggesting that, at least by the age of five, children form consistent first impressions of others [13, 42]. In future research, it would be interesting to investigate whether parents talk to even younger children about the apparent character traits of novel individuals and to examine in what ways parental conversations with their children change over time. Studies with younger children would help disambiguate whether parents create face-trait mappings in their children as well as reinforcing the face-trait mappings their children already possess.

While these data highlight the wealth of social information available to children regarding how appearance relates to character, they do not provide evidence that these types of social experiences play a causal role in children’s developing first impressions. In future research, it would be interesting to experimentally manipulate how an experimenter talks to children about faces and then measure whether this influences children’s first impressions on a judgment task. The types of parental conversation recorded in this study could provide a useful starting point for developing such a manipulation.

Of further interest is the finding that, in both studies, parents and children spoke about the emotions of the individuals depicted as well as their apparent character traits. This was the case even though participants had been given no prompting to do so and the stimuli used in both studies were designed to be emotionally neutral [1, 37]. These findings underscore previous research showing a tight connection between emotional expression and trait judgments [43, 44]. One possible explanation for this connection is offered by the ‘emotional overgeneralisation hypothesis’. According to this hypothesis, individuals whose facial features subtly resemble smiles tend to be judged more trustworthy than individuals whose facial features subtle resemble frowns [45, 46]. An alternative explanation is that the extent to which faces used in first impressions research are truly ‘emotionally neutral’ may have been overestimated in previous work. Developmental work that seeks to investigate first impressions in the absence of emotional cues may wish to control their stimuli more closely [14, 47].

A further interesting aspect of our findings relates to parents’ explicit rejection of judging others based on their appearance. Even though all parents and their children engaged in at least some discussion about the apparent character traits of the individuals depicted, parents tended to state that it was inappropriate to judge others based on their appearance. These results must be interpreted with caution because of the modest sample size and the lack of anonymity in parents’ responses. Future research may consider collecting larger samples in more anonymous settings, for example, through online data collection. Nevertheless, our data do suggest some interesting possibilities for further work. If future work seeks to modify the ways in which parents teach their children about first impressions, our research suggests it will be important to develop interventions that target their actual teaching behaviour rather than merely their attitudes about teaching.

Previous research has shown that trait judgments emerge early in development [13, 42, 48] and suggested that learning plays a role in the acquisition of these judgments [18, 22]. The research reported here moves beyond previous research by starting to investigate *how* this learning takes place. In doing so, it opens up a number of interesting avenues for future research. For example, in future work it will be important to explore how conversations between parents and children differ depending on the nature of the faces depicted. In these studies, we presented parents and children with picture books containing images of Caucasian individuals. In future research, it will be important to vary the ethnicity of the individuals depicted. Exploring how parental conversation varies depending on the group membership of the individuals depicted would help integrate the study of first impressions with research on stereotyping and prejudice. Previous research has shown that Caucasian parents are often reluctant to discuss ‘race’ and racism with their children [49]. In this context, it would be very interesting to determine whether trait discussion could capture implicit biases in parental conversation.

Whereas we chose to focus on verbal behaviour to understand parent-child interaction, it will be important for future research to investigate how the non-verbal behaviour displayed by parents influences children’s inferences about traits. Non-verbal behaviour such as emotional expression and gesturing have been shown to impact children’s social judgements [18, 50]. Future research could analyse the valence of parents’ initial expression when each face is revealed and how it varies according to the particular face depicted.

Future work may also investigate parent-child conversation in the absence of any instruction to talk about the faces depicted. One route by which to do this would be to give parents and children a seemingly unrelated task, such as memorising the faces, and measuring incidental conversation about traits. Another route by which to achieve this would to be to analyse corpus data for evidence of naturally occurring conversations about face-trait mappings.

Finally, in future research it would be interesting to investigate how the composition and cultural background of parent-child dyads influences conversations about the apparent traits and emotions of the individuals depicted. Previous research suggests that mothers may be more likely to make references to emotions, and to use causal explanatory language when referring to emotions, than fathers [51]. Furthermore, there are systematic cultural differences in the first impressions that individuals form which may manifest themselves in different styles of parent-child interaction [52, 53].

The study of first impressions is becoming increasingly prominent within the developmental literature. Recent research has investigated the developmental origins [14, 54] and behavioural consequences [15] of first impressions among children. We contributed to this work by exploring one of the developmental mechanisms through which first impressions may be acquired and/or reinforced. Our data suggest that parental conversation is one plausible mechanism through which first impressions could be learned [18].

## Supporting information

S1 Appendix Study 1 mixed models: Tables A-C. (PDF)

S2 Appendix Study 1 trait and emotion terms: Tables A-D. (PDF)

S3 Appendix Study 2 mixed models: Tables A-C. (PDF)

S4 Appendix Study 2 trait and emotion terms: Tables A-D. (PDF)
