# Supplementary material for: Parents reinforce the formation of first impressions in conversation with their children
Source: PLoS One. 2021 Aug 13;16(8):e0256118. doi: 10.1371/journal.pone.0256118 (PMC8362939; doi:10.1371/journal.pone.0256118)
Supplement: S1 Appendix — (PDF) [file pone.0256118.s001.pdf]

S1 Appendix

Study 1: Mixed Models. Tables A-C

Table A

Study 1: Linear mixed models fixed and random effects comparing the numbers of words spoken by parents between conditions (left) and children between conditions (right).

	Parent Word Count			Child Word Count		
Predictors	*Estimates*	*CI*	*p*	*Estimates*	*CI*	*p*
Intercept (Low Trustworthiness)	78.04	57.20 – 98.88	**<0.001**	34.42	23.68 – 45.15	**<0.001**
High Competence	5.13	-7.12 – 17.37	0.412	-9.42	-17.87 – -0.96	**0.029**
High Trustworthiness	1.71	-10.53 – 13.95	0.784	-0.63	-9.08 – 7.83	0.885
Low Competence	0.46	-11.78 – 12.70	0.942	2.75	-5.70 – 11.20	0.524
**Random Effects**						
σ^2	468.21			223.24		
τ_{00}	2244.58 participant_id			497.10 participant_id		
ICC	0.83			0.69		
N	24 participant_id			24 participant_id		
Observations	96			96		
Marginal R^2 / Conditional R^2	0.001 / 0.828			0.028 / 0.699		

Table B
Study 1: Generalised linear mixed effect models, odds ratios and random effects comparing likelihood of parents (left) and children (right) using trait terms between conditions.

	Parent Traits – Binomial			Child Traits - Binomial		
Predictors	*Odds Ratios*	*CI*	*p*	*Odds Ratios*	*CI*	*p*
Intercept (Low Trustworthiness)	2.47	0.38 – 16.23	0.346	1.41	0.27 – 7.36	0.681
High Competence	0.32	0.05 – 1.87	0.205	0.35	0.07 – 1.91	0.227
High Trustworthiness	0.47	0.08 – 2.65	0.391	0.35	0.07 – 1.91	0.227
Low Competence	0.21	.03 – 1.34	0.099	0.17	0.03 – 1.03	0.054
**Random Effects**						
σ^2	3.29			3.29		
τ_{00}	10.58 participant_id			7.84 participant_id		
ICC	0.76			0.70		
N	24 participant_id			24 participant_id		
Observations	96			96		
Marginal R^2 / Conditional R^2	0.023 / 0.768			0.035 / 0.715		

Table C
Study 1: Generalised linear mixed effect models, odds ratios and random effects comparing likelihood of parents (left) and children (right) using emotion terms between conditions.

	**Parent Emotions – Binomial**			**Child Emotions - Binomial**		
Predictors	*Odds Ratios*	*CI*	*p*	*Odds Ratios*	*CI*	*p*
Intercept (Low Trustworthiness)	0.77	0.20 – 3.02	0.708	0.46	0.13 – 1.61	0.224
High Competence	0.75	0.17 – 3.34	0.704	0.30	0.06 – 1.47	0.138
High Trustworthiness	0.75	0.17 – 3.34	0.704	2.17	0.52 – 9.14	0.290
Low Competence	0.41	0.09 – 1.92	0.257	0.57	0.13 – 2.51	0.459
**Random Effects**						
σ^2	3.29			3.29		
τ_{00}	4.43 $_{participant\_id}$			2.92 $_{participant\_id}$		
ICC	0.57			0.47		
N	24 $_{participant\_id}$			24 $_{participant\_id}$		
Observations	96			96		
Marginal R^2 / Conditional R^2	0.014 / 0.580			0.079 / 0.512		
