# Supplementary material for: Parents reinforce the formation of first impressions in conversation with their children
Source: PLoS One. 2021 Aug 13;16(8):e0256118. doi: 10.1371/journal.pone.0256118 (PMC8362939; doi:10.1371/journal.pone.0256118)
Supplement: S2 Appendix — (PDF) [file pone.0256118.s002.pdf]

S2 Appendix

Study 1 Trait and Emotion Terms: Tables A-D

Table A

Parent Trait Terms

Face	Term	Frequency
High Trustworthiness	Nice	9
	Kind	5
	Friendly	3
	Friendlier	2
	Naughty	2
	Sporty	2
	Good	1
	Grumpy	1
	Helpful	1
	Mean	1
Low Trustworthiness	Boring	6
	Nice	6
	Friendly	4
	Bad	3
	Good	3
	Grumpy	3
	Kind	3
	Meanie	2
	Scary	2
	Creative	1
	Friendlier	1
	Intimidating	1
	Nasty	1
	Naughty	1
	Sensible	1
	Sporty	1
High Competence	Nice	10
	Adventurous	3
	Brave	3
	Cheerful	3
	Good	3
	Intelligent	3
	Kind	3
	Mean	3
	Friendly	2
	Grumpy	2
	Nasty	2

Table A - Continued		
High Competence	Caring	1
	Helpful	1
	Scary	1
	Sporty	1
Low Competence	Nice	7
	Kind	5
	Evil	4
	Friendly	4
	Good	2
	Grumpy	2
	Scary	2
	Depressed	1
	Depression	1
	Mean	1
	Nosey	1

Table B

Child Trait Terms

Face	Term	Frequency
High Trustworthiness	Nice	4
	Kind	2
	Friendly	1
	Good	1
	Naughty	1
	Sporty	1
Low Trustworthiness	Boring	4
	Smart	3
	Bad	1
	Good	1
	Grump	1
	Grumpy	1
	Meanie	1
	Nasty	1
	Naughty	1
	Nice	1
	Scary	1
	Sensible	1
	Sporty	1
High Competence	Nice	4
	Adventurous	1
	Brave	1
	Cheerful	1
	Good	1
	Grumpy	1
	Mean	1
	Scary	1
Low Competence	Depressed	2
	Evil	2
	Scary	2
	Bad	1
	Good	1
	Grumpy	1
	Kind	1
	Nice	1
	Nosey	1

Table C

Parent Emotion & Expression Terms

Face	Term	Frequency
High Trustworthiness	Happy	15
	Sad	5
	Smile	3
	Smiling	2
	Angry	1
	Happier	1
	Straight Face	1
Low Trustworthiness	Happy	10
	Sad	6
	Angry	5
	Moody	2
	Cross	1
	Frowning	1
	Joyful	1
High Competence	Happy	8
	Sad	6
	Crosser	3
	Smiling	3
	Angry	1
	Happier	1
	Smile	1
	Smiley	1
	Straight face	1
Low Competence	Happy	7
	Sad	6
	Cross	1
	Nervous	1
	Sadder	1
	Scared	1
	Smiling	1
	Surprised	1
	Tearful	1

Table D

Child Emotion & Expression Terms

Face	Term	Frequency
High Trustworthiness	Happy	9
	Cross	1
	Happier	1
	Smile	1
	Smiley	1
	Smiling	1
	Straight Face	1
Low Trustworthiness	Angry	4
	Sad	3
	Cross	1
	Frowning	1
	Joyful	1
	Moody	1
High Competence	Crosser	2
	Cross	1
	Happy	1
	Smile	1
	Smiling	1
	Straight Face	1
Low Competence	Sad	3
	Happy	2
	Frowny	1
	Sadder	1
	Smiling	1
