# Supplementary material for: Parents reinforce the formation of first impressions in conversation with their children
Source: PLoS One. 2021 Aug 13;16(8):e0256118. doi: 10.1371/journal.pone.0256118 (PMC8362939; doi:10.1371/journal.pone.0256118)
Supplement: S3 Appendix — (PDF) [file pone.0256118.s003.pdf]

S3 Appendix

Study 1: Mixed Models. Tables A-C

Table A

Study 2: Linear mixed models fixed and random effects comparing the numbers of words spoken by parents between conditions (left) and children between conditions (right)..

	Parent Word Count			Child Word Count		
Predictors	*Estimates*	*CI*	*p*	*Estimates*	*CI*	*p*
(Intercept) Low Trustworthiness	76.62	56.99 – 96.26	**<0.001**	25.62	19.64 – 31.61	**<0.001**
High Intelligence	4.79	-12.02 – 21.60	0.576	-2.17	-7.74 – 3.40	0.446
High Trustworthiness	-3.46	-20.27 – 13.35	0.687	-2.58	-8.15 – 2.99	0.363
Low Intelligence	-7.29	-24.10 – 9.52	0.395	-2.42	-7.99 – 3.15	0.395
**Random Effects**						
σ^2	882.56			96.90		
τ_{00}	1526.20 participant_id			127.08 participant_id		
ICC	0.63			0.57		
N	24 participant_id			24 participant_id		
Observations	96			96		
Marginal R^2 / Conditional R^2	0.008 / 0.637			0.005 / 0.569		

Table B
Study 2: Generalised linear mixed effect models, odds ratios and random effects comparing likelihood of parents (left) and children (right) using trait terms between conditions.

	**Parent Traits – Binomial**			**Child Traits - Binomial**		
Predictors	*Odds Ratios*	*CI*	*p*	*Odds Ratios*	*CI*	*p*
(Intercept) Low Trustworthiness	1.27	0.41 – 3.97	0.683	1.25	0.41 – 3.77	0.697
High Intelligence	0.23	0.05 – 0.98	**0.047**	0.31	0.08 – 1.25	0.099
High Trustworthiness	0.49	0.12 – 1.93	0.308	0.39	0.10 – 1.55	0.183
Low Intelligence	1.00	0.26 – 3.87	1.000	0.63	0.16 – 2.41	0.500
**Random Effects**						
σ^2	3.29			3.29		
τ_{00}	2.29 participant_id			2.01 participant_id		
ICC	0.41			0.38		
N	24 participant_id			24 participant_id		
Observations	96			96		
Marginal R^2 / Conditional R^2	0.064 / 0.448			0.038 / 0.403		

Table C
Study 2: Generalised linear mixed effect models, odds ratios and random effects comparing likelihood of parents (left) and children (right) using emotion terms between conditions.

	**Parent Emotions – Binomial**			**Child Emotions - Binomial**		
Predictors	*Odds Ratios*	*CI*	*p*	*Odds Ratios*	*CI*	*p*
Intercept (Low Trustworthiness)	0.67	0.10 – 4.31	0.670	0.65	0.09 – 4.83	0.670
High Intelligence	7.62	1.04 – 55.67	**0.045**	3.57	0.54 – 23.87	0.189
High Trustworthiness	19.11	2.05 – 177.77	**0.010**	23.51	2.17 – 254.63	**0.009**
Low Intelligence	3.30	0.53 – 20.62	0.202	1.00	0.16 – 6.06	1.000
**Random Effects**						
σ^2	3.29			3.29		
τ_{00}	10.85 $_{participant\_id}$			13.07 $_{participant\_id}$		
ICC	0.77			0.80		
N	24 $_{participant\_id}$			24 $_{participant\_id}$		
Observations	96			96		
Marginal R^2 / Conditional R^2	0.078 / 0.785			0.094 / 0.818		
