# Supplementary material for: Parents reinforce the formation of first impressions in conversation with their children
Source: PLoS One. 2021 Aug 13;16(8):e0256118. doi: 10.1371/journal.pone.0256118 (PMC8362939; doi:10.1371/journal.pone.0256118)
Supplement: S4 Appendix — (PDF) [file pone.0256118.s004.pdf]

S4 Appendix

Study 2 Trait and Emotion Terms: Tables A-D

Table A

Parent Trait Terms

Face	Term	Frequency
High Trustworthiness	Nice	5
	Serious	4
	Cool	2
	Goody	2
	Scary	2
	Awesome	1
	Bad	1
	Crazy	1
	Good	1
	Kind	1
	Nasty	1
Low Trustworthiness	Scary	9
	Naughty	6
	Baddy	3
	Mean	3
	Clever	2
	Good	2
	Goody	2
	Mad	2
	Nice	2
	Cleverer	1
	Grumpy	1
	Lazy	1
High Intelligence	Nice	5
	Bad	4
	Friendly	4
	Scary	3
	Good	2
	Kind	2
	Naughty	2
	Clever	1
	Crazy	1
	Serious	1
	Shifty	1
Low Intelligence	Crazy	8
	Grumpy	4
	Good	3
	Lazy	3
	Goody	2

Table A - Continued		
Low Intelligence	Silly	2
	Bad	1
	Mad	1
	Naughty	1
	Scary	1
	Serious	1
	Trustworthy	1
	Untrustworthy	1

Table B

Child Trait Terms

Face	Term	Frequency
High Trustworthiness	Serious	3
	Awesome	2
	Nice	2
	Bad	1
	Cool	1
	Goody	1
Low Trustworthiness	Goody	2
	Scary	2
	Bad	1
	Baddy	1
	Clever	1
	Daring	1
	Grumpy	1
	Lazy	1
	Mad	1
	Mean	1
	Naughty	1
	Suspicious	1
High Intelligence	Nice	2
	Bad	1
	Good	1
	Kind	1
	Naughty	1
	Scary	1
Low Intelligence	Grumpy	6
	Crazy	3
	Good	2
	Lazy	2
	Goody	1
	Mad	1
	Naughty	1
	Scary	1
	Silly	1

Table C

Parent Emotion & Expression Terms

Face	Term	Frequency
High Trustworthiness	Happy	17
	Sad	16
	Bored	5
	Confused	3
	Smile	3
	Angry	1
	Frown	1
	Miserable	1
	Smiles	1
	Smiling	1
	Unhappy	1
Low Trustworthiness	Sad	13
	Happy	11
	Angry	10
	Bored	2
	Smiling	2
	Unhappy	2
	Confused	1
	Cross	1
High Intelligence	Happy	15
	Sad	8
	Smile	5
	Confused	4
	Happier	4
	Angry	3
	Smiling	2
	Scowls	1
	Straight Faced	1
Low Intelligence	Sad	22
	Happy	12
	Confused	3
	Angry	2
	Cross	2
	Nervous	2
	Unsure	1

Table D

Child Emotion & Expression Terms

Face	Term	Frequency
High Trustworthiness	Sad	7
	Happy	6
	Bored	3
	Confused	3
	Frown	1
	Miserable	1
Low Trustworthiness	Sad	5
	Angry	4
	Bored	1
	Confused	1
	Cross	1
	Happier	1
	Happy	1
	Sadder	1
	Smiling	1
High Intelligence	Happy	8
	Confused	2
	Sad	2
	Smile	2
	Angry	1
Low Intelligence	Sad	6
	Happy	2
	Nervous	2
	Angry	1
	Confused	1
	Cross	1
	Smile	1
